# The Rehabilitation Tailor: Applying Personalized Medicine to Cancer Recovery

**DOI:** 10.3389/fgwh.2022.914302

**Published:** 2022-07-12

**Authors:** Giulia Bongiorno, Helena Biancuzzi, Francesca Dal Mas, Rym Bednarova, Luca Miceli

**Affiliations:** ^1^Friuli Riabilitazione, Roveredo in Piano, Italy; ^2^Pain Medicine, IRCCS National Cancer Institute of Aviano, Aviano, Italy; ^3^Department of Management, Ca Foscari University of Venice, Venice, Italy; ^4^Pain Medicine, Hospital of Latisana, Latisana, Italy

**Keywords:** cancer rehabilitation, physiotherapy, breast cancer, fitness activity, women

## Introduction

Precision medicine represents one of the frontiers in oncological treatments as it allows, mainly through the study of patients' genetic profiles, to build a treatment path that is as personalized as possible (1).

The new challenge is to create a rehabilitation program that can be tailored to the patient's needs, to be carried on at a distance, allowing the patient to be educated, and be followed up in the oncological recovery.

The National Cancer Institute of Aviano, Italy, is starting a new program called “Rehabilitation Tailor,” aiming to offer a personalized rehabilitation journey to patients with cancer by leveraging the most modern technologies. The institute is not new to creating tailored educational programs for oncological patients, including the Oncology in Motion experience (2, 3) to stimulate patients with cancer in co-producing their recovery through fitness activities at a distance and the Doctor @ Home telemedicine journey (4), involving continuous co-production and co-learning paths between clinicians and patients.

## The Rehabilitation Tailor Program in Breast Cancer

**Figure 1:**
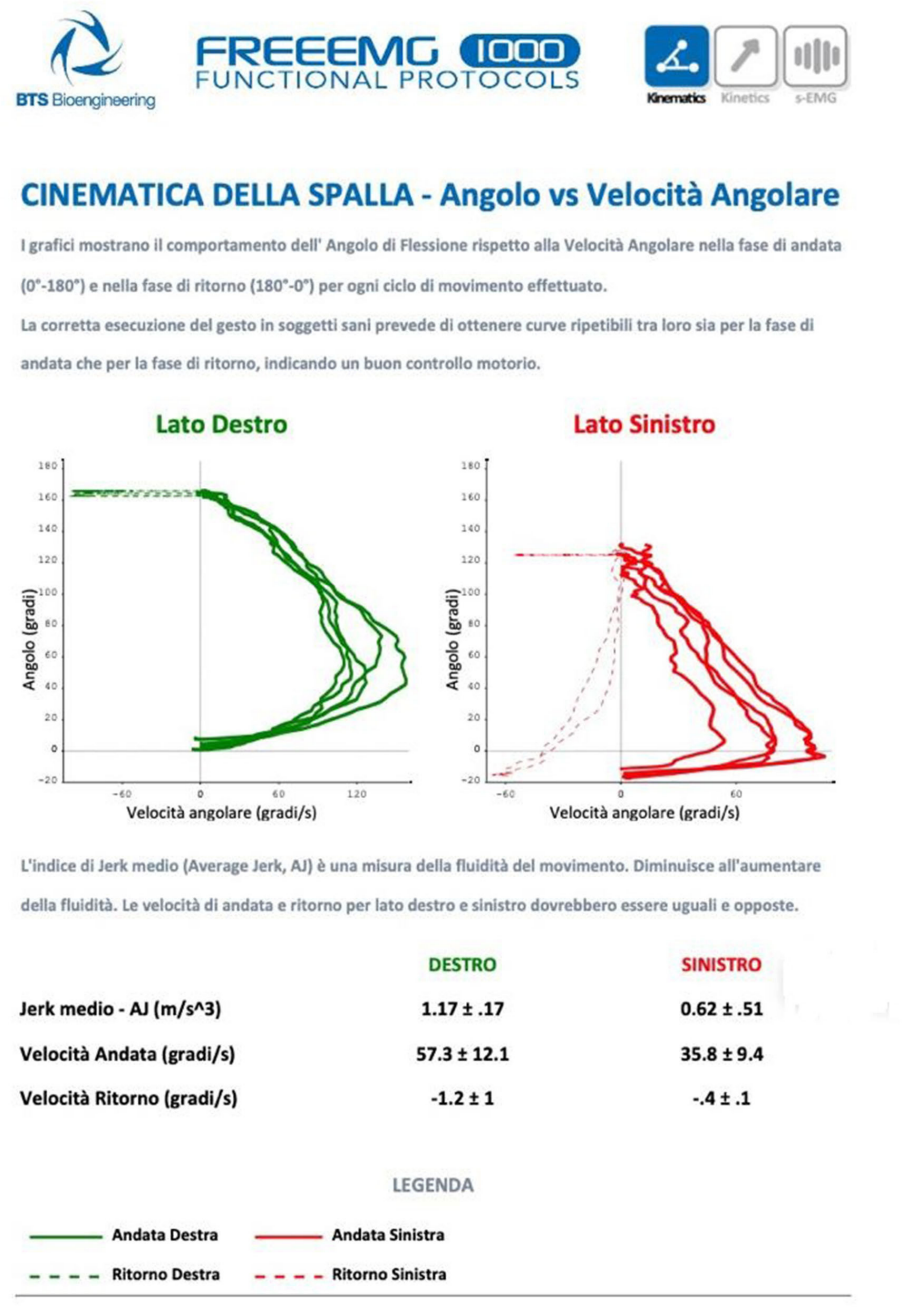
The graphs show the behavior of the bending angle concerning the angular velocity in the outward and return phases for each cycle of movement performed. The correct execution of the gesture in healthy patients provides repeatable curves for both the outward and return phases, indicating good motor control.

The project involves the creation of a Personal Rehabilitation Electronic Record (PRER), which is then to be shared with the patient and all the professionals supporting her in the healing process, including the physiotherapist in charge (Figure 1).

This personalized path allows to build a specific osteoarticular and muscular profile as a guide for setting up correct physiotherapy and, if necessary, an appropriate muscle strengthening program, educating the patients in the co-production of their cancer rehabilitation (5). More importantly, the path allows periodic monitoring (about two times a year) and follow-up with detailed numerical indices of the progress (range of movement, jerk index, and the percentage contribution of each muscle to the investigated movement), especially when the patients need to perform their rehabilitation treatments directly from their homes.

The path includes patients who had breast operations, for whom the need for a rehabilitation process is recognized, and a non-invasive assessment on several levels to create a scorecard.

Several referenced indexes are calculated and validated, as described below, with the use of technological devices like sensors. The electronic record is then shared with the patient, with a multidisciplinary approach involving a physiotherapist and a pain therapist.

**Figure 2:**
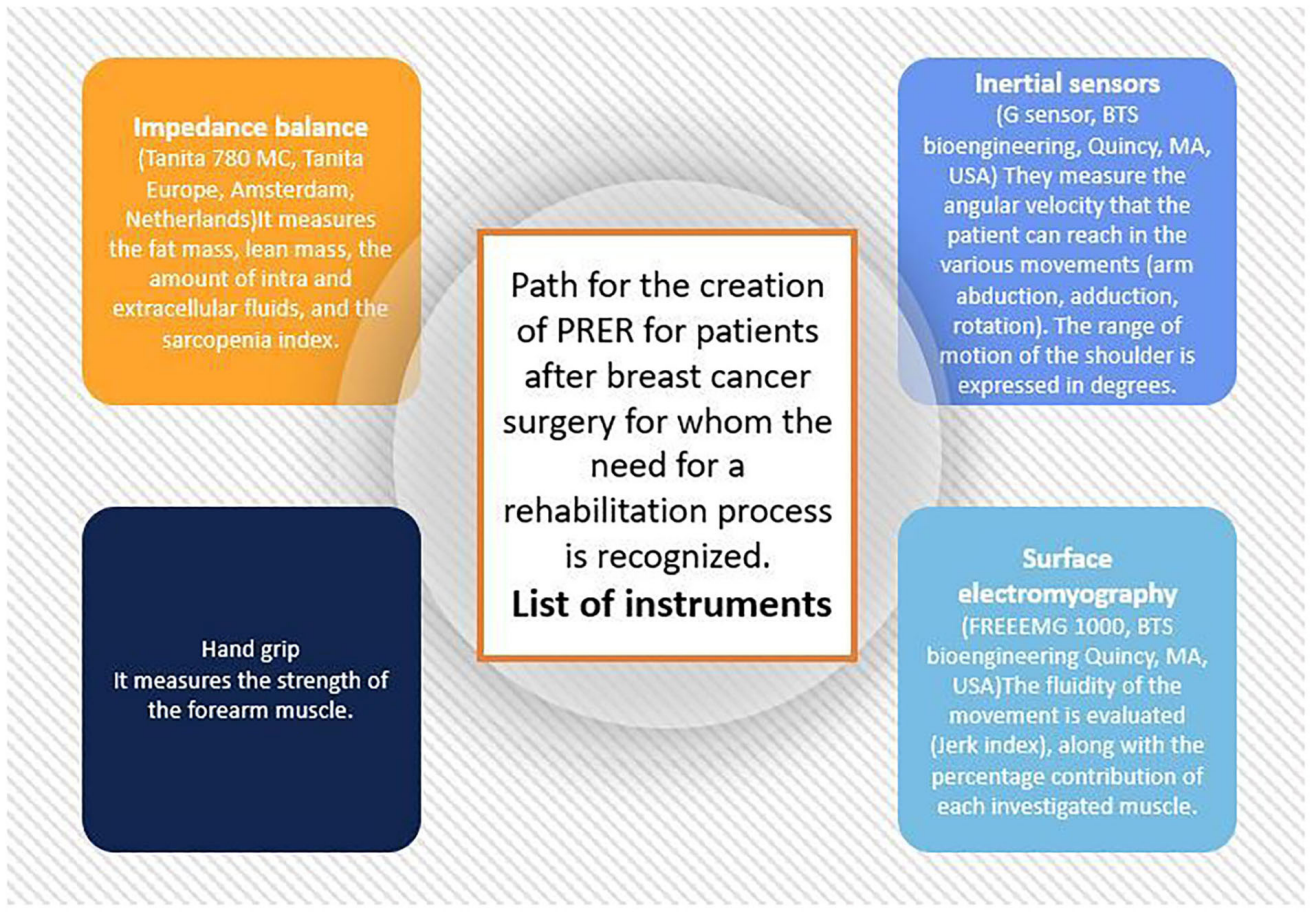
Path for the creation of PRER for patients after breast surgery for whom the need for a rehabilitation process is recognized.

The assessment and creation of the PRER stands as follows. Through the use of inertial sensors (G sensor, BTS bioengineering, Quincy, MA, USA) the range of movement of the shoulder is expressed in degrees, and the angular velocity that the patient can reach in the various movements (arm abduction, adduction, and rotation). Subsequently, with surface electromyography (Freeemg 1000, BTS bioengineering Quincy, MA, USA), the fluidity of the movement is evaluated (Jerk index), and the percentage contribution of each muscle investigated to the movement is studied, as well as the effort made by the patient in muscular terms to reach certain angles of movement, which is consistent with other experiences described in the literature (6). A final assessment then involves evaluating a possible decrease in muscle mass and strength, the so-called sarcopenia, which often stands as a consequence of oncological treatments (7). An impedance balance is used to evaluate the fat mass, lean mass, the amount of intra and extracellular fluids, and the sarcopenia index (Tanita 780 MC, Tanita Europe, Amsterdam, Netherlands). An instrument called “hand grip” measures the strength of the forearm muscles. With these indices, it is possible to obtain the quality index of the upper limb muscle of the patients (8). Finally, in case of shoulder pain that limits the correct rehabilitation process, the patient is followed by the pain therapist, using peripheral nerve neuromodulation techniques, mainly of the suprascapular nerve (9), trying as much as possible to limit the use of drugs, such as opioids. The instruments used, as well as their aims, are reported in the following Figure 2.

## Conclusions

The Rehabilitation Tailor Program and the PRER were generated, thanks to the assessment for allowing to set up a possible course of physical activity and a muscle strengthening to be carried on at home. The use of modern technologies, like sensors and devices, plays a crucial role as knowledge-translation facilitators to educate the patients in carrying on the required rehabilitation tasks, measuring their progress, and allowing the connection between the institute's staff (including oncologists and pain medicine specialists) and the preferred support clinicians, like physiotherapists. Moreover, the amount of data collected may support the progress of rehabilitation programs in general terms, allowing the detailed measurement of the recovery outcomes and stimulating the promising link between fitness activity and cancer rehabilitation (10).

The Rehabilitation Tailor Program is a project that stands as a step forward in the translational application of personalized medicine in oncological care, and its potential is to be applied to all cancer follow-ups and recovery.

## Ethics Statement

Written informed consent was obtained from the individuals for the publication of any potentially identifiable images or data included in this article.

## Author Contributions

GB and LM conceived the idea of the study and took care of data collection and analysis. HB, FD, and LM took care of the drafting of the article. All authors reviewed, read, and approved the final version of the manuscript.

## Conflict of Interest

The authors declare that the research was conducted in the absence of any commercial or financial relationships that could be construed as a potential conflict of interest.

## Publisher's Note

All claims expressed in this article are solely those of the authors and do not necessarily represent those of their affiliated organizations, or those of the publisher, the editors and the reviewers. Any product that may be evaluated in this article, or claim that may be made by its manufacturer, is not guaranteed or endorsed by the publisher.
